# Acidification-dependent suppression of *C. difficile* by pathogenic and commensal enterococci

**DOI:** 10.1128/iai.00159-26

**Published:** 2026-05-13

**Authors:** Holly R. Neubauer, Ibukun M. Ogunyemi, Alicia K. Wood, Angus Johnson, Avi Z. Stern, Zainab Sikander, Lesly-Hannah Gutierrez, Addelis A. Agosto, Peter T. McKenney

**Affiliations:** 1 Binghamton Biofilm Research Center, Department of Biological Sciences, Binghamton University, SUNY 171431, Binghamton, New York, USA; University of Illinois Chicago, Chicago, Illinois, USA

**Keywords:** microbiota, *Enterococcus*, VRE, *C. difficile*

## Abstract

*Clostridioides difficile* and vancomycin-resistant *Enterococcus faecium* (VRE) are commonly co-isolated from hospitalized patients. We sought to develop a co-culture biofilm model to characterize interactions between these two opportunistic pathogens. Upon growth in biofilm-promoting media containing added glucose, fructose, or trehalose, VRE produces sufficient acid to lower the pH and inhibit growth of *C. difficile*. We found this effect depended on the carbon source, and that acidification by VRE was necessary and sufficient to suppress *C. difficile* growth in liquid medium and in cecal content extracts from germ-free mice. VRE frequently dominates the intestine of patients administered antibiotics, which can predispose to the development of *C. difficile* infection. We reasoned that it may be possible to suppress *C. difficile* growth during co-infection with VRE by supplementing the mouse diet with a fermentable sugar. A VRE-dominated gut microbiota may convert the sugar to acid, lower the pH, and reestablish colonization resistance to *C. difficile*. Supplementation of the diet of VRE-colonized mice with high levels of fructose neither resulted in a lower pH nor did it prevent colonization by *C. difficile*. Taken together, these data suggest that VRE can suppress growth of *C. difficile* by organic acid production in a carbon source-dependent manner *in vitro*; however, the mammalian intestine may require sophisticated approaches to lower pH therapeutically.

## INTRODUCTION

High population diversity in the gut microbiota is generally correlated with health across gastrointestinal diseases. Treatment with antibiotics can disrupt the gut microbiota to the point that a single species can expand and dominate the gut, making up a high percentage of the reads in a 16S microbiome sequencing data set. Treatment with broad-spectrum antibiotics can lead to expansion of VRE (1) and domination of the gut microbiota by the genus *Enterococcus*. This was observed in humans (2) and mice (3). This domination occurs in hematopoietic stem cell transplant patients where a low diversity microbiota is correlated with mortality (2, 4). Vancomycin-resistant *Enterococcus faecium* (VRE) is a rising clinical concern that causes difficult-to-treat systemic infections. However, it is often forgotten that *E. faecium* is also a common member of the human gut microbiota, a lactic acid bacterium, and a commonly used probiotic in humans and agricultural animal production (5–7).

The majority of studies suggest that patients with a low-diversity gut microbiota following antibiotic treatment are also at elevated risk for *Clostridioides difficile* infection (CDI). Colonization of the gut with VRE and the genus *Enterococcus* has been correlated with increased risk of CDI in hospitalized human patients (8–10). *Enterococcus* species in the gut microbiota were predicted to enhance CDI risk in mathematical modeling that combined data from human and mouse infections (11). In mouse models, genus *Enterococcus* was also correlated with increased persistence of *C. difficile* in the gut (12) and increased toxin production and virulence under high dietary zinc conditions (13). One other study described the correlation of genus *Enterococcus* abundance with attenuation of *C. difficile* virulence (14). It is possible that strain diversity may contribute to differing results as some commensal enterococci suppress *C. difficile* growth *in vitro* (15).

Two direct tests of the effects of *Enterococcus* colonization on the CDI mouse model reported higher toxin titer and pathology (10, 16). The mechanism of enhancement of CDI severity in mice was linked to metabolic cross-talk between *E. faecalis* OG1RF and *C. difficile*, in which arginine supplementation was sufficient to reduce toxin production and pathology, without significantly affecting colonization by either organism (10). Furthermore, *E. faecalis* benefits from the release of host heme caused by *C. difficile* toxin-mediated damage in mouse models of CDI (17).

Both *C. difficile* and enterococci form biofilms (18, 19). In both species, biofilms contribute to antibiotic tolerance (20, 21) and may be a reservoir for infection recurrence in CDI (22, 23). This study began as an attempt to establish an *in vitro* co-culture biofilm model of VRE and *C. difficile*. We quickly noticed that the byproducts of primary metabolism produced by VRE negatively affected *C. difficile* growth. Here, we have established that VRE and commensal enterococci are capable of acidifying unbuffered bacterial growth media below a pH that is growth inhibitory to *C. difficile* and other commensal and opportunistic clostridia. Under these conditions, we show that acidification of a carbon source is necessary and sufficient for inhibition of *C. difficile* and clostridia growth. These data suggest that in conditions where lactic acid bacteria, such as VRE, dominate the local environment, alteration of acid production and pH levels in a carbon source-dependent manner affects the viability of *C. difficile* in co-culture *in vitro*. Finally, we modeled the effects of carbon source control in VRE-dominated mice by supplementing the diet with high levels of fructose followed by *C. difficile* infection. Fructose supplementation did not affect *C. difficile* CFU levels in VRE co-infected mice. In our mouse model of co-infection, fructose supplementation alone was not sufficient to alter *C. difficile* colonization, suggesting that more complex interventions may be necessary to reproduce the *in vitro* phenomenon.

## RESULTS

### Glucose metabolism inhibits *C. difficile* growth in co-culture with VRE

**Fig 1:**
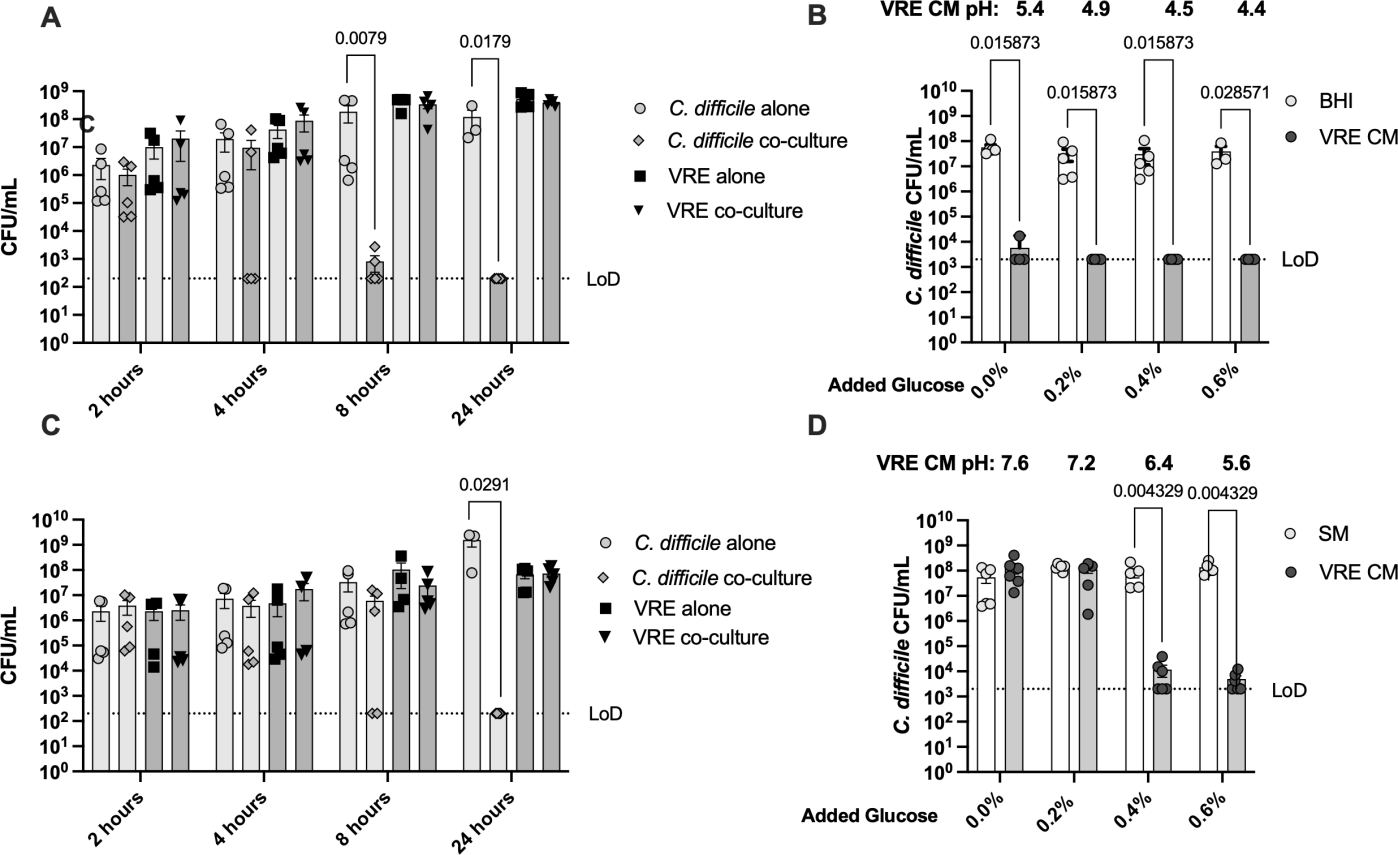
*C. difficile* is inhibited by VRE during co-culture in excess glucose. (**A**) Time course CFUs of *C. difficile* and VRE alone and in co-culture in BHI + 0.4% glucose (0.6% total glucose). Data are combined from two independent experiments, *n* = 3–5 biological replicates per time point, Mann-Whitney test of alone vs co-culture. (**B**) VRE was grown for 48 h in BHI + 0%, 0.2%, 0.4%, or 0.6% additional glucose, then filter sterilized to create conditioned media (VRE CM), which was then inoculated with *C. difficile*. The average pH of the VRE CM is shown above the graph. Data are combined from two independent experiments, *n* = 3–5 biological replicates per time point, Mann-Whitney test. (**C**) Time course CFUs of *C. difficile* and VRE alone and in co-culture in SM + 0.6% glucose. Data are combined from two independent experiments, *n* = 3–5 biological replicates per time point, Mann-Whitney test of alone vs co-culture. (**D**) VRE was grown for 48 h in SM + 0%, 0.2%, 0.4%, or 0.6% additional glucose. CM was filter sterilized to create VRE CM, and the average pH of the VRE CM is shown above the graph. It was then inoculated with mid-log *C. difficile* and grown for 48 h. Data are combined from two independent experiments, *n* = 5–6 biological replicates per time point, Mann-Whitney test.

We began this work as an attempt to establish a dual-species *in vitro* liquid biofilm model for VRE and *C. difficile*. Typically for Gram-positive bacteria, glucose is added to the culture media to a final concentration of 0.2%–1%, which promotes attachment and production of exopolysaccharide matrix (20, 24–27). When we performed a pilot experiment of liquid co-culture growth in Supplemented Brain Heart Infusion (BHI) broth with 0.4% added glucose (total glucose = 0.6%), we found a significant decrease in *C. difficile* growth at 8 and 24 h post-inoculation when in co-culture with VRE (Fig. 1A). We observed a similar elimination of *C. difficile* when co-cultured with VRE in Sporulation Media (SM), which lacks an added carbon source, when it was supplemented with 0.4% glucose or greater in co-culture (Fig. 1C).

Next, to determine if the inhibitory factor produced by VRE is soluble and filterable, we generated conditioned media by growing VRE to exhaustion (OD600 ~1.8) and filtered it through a 0.22-μm filter. To determine if the inhibition was dose-dependent, we generated VRE conditioned media with escalating levels of added glucose. The conditioned medium was then inoculated with *C. difficile* and grown for 48 h. We found that increasing the concentration of glucose results in inhibition of *C. difficile* growth and that the inhibitory factor is soluble and filterable in both BHI and SM (Fig. 1B and D). Enterococci are closely related to lactic acid bacteria and are known to produce large amounts of acid when cultured in glucose (28). We tested the pH of the VRE conditioned media and found that pH is inversely correlated with glucose concentration. In BHI conditioned media, the starting pH was <5.3 for all tested glucose concentrations, which did not support *C. difficile* growth (Fig. 1B). BHI contains 0.2% glucose as formulated and is unbuffered, likely accounting for the reduced pH. In conditioned SM with 0.4% added glucose and an average starting pH of 6.4, *C. difficile* growth was not supported (Fig. 1D). These data suggest an inhibitory pH threshold of around six for *C. difficile* in VRE conditioned media. We note that *C. difficile* itself is capable of lowering medium pH when in monoculture in both SM (pH 6.5) and BHI (pH 5.7) with added glucose; however, VRE lowers pH by an additional unit in both media (Fig. S1). When fructose is added to co-cultures during stationary phase, *C. difficile* CFUs were significantly reduced along with a corresponding drop in pH (Fig. S2). These data suggest that inhibition is not limited to the exponential growth phase. This effect can be bactericidal as resuspending late log-phase *C. difficile* in VRE-conditioned high glucose media for 2 h resulted in a significant reduction in *C. difficile* (Fig. S3). To further test if acidification is necessary for inhibition of *C. difficile* growth in VRE-conditioned media, we grew VRE to exhaustion in SM ± 0.6% glucose with 100 mM of HEPES, MOPS, or PIPES buffer (Fig. S4). Buffering with MOPS resulted in a rise in the pH of the conditioned medium from 5.7 to 6.1, which resulted in a partial rescue of *C. difficile* growth. Taken together, these data suggest that in glucose-rich media, VRE produces organic acids that lower pH and inhibit *C. difficile*.

**Fig 2:**
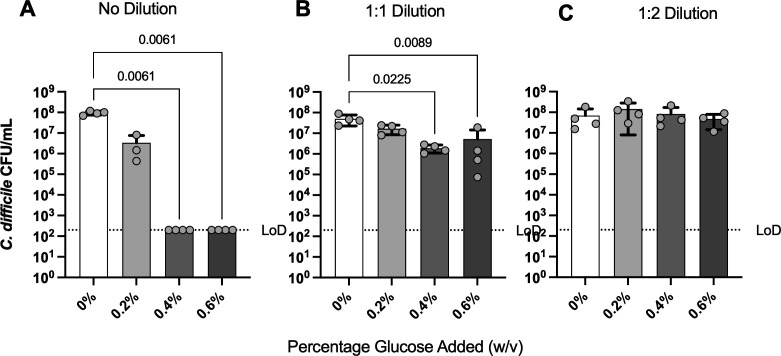
Inhibition by VRE-conditioned media is not caused by nutrient limitation. VRE conditioned sporulation media (SM) were generated with increasing concentrations of added glucose, filter sterilized, then kept undiluted (**A**) or diluted 1:1 (**B**) or 1:2 (**C**) with reduced PBS before inoculation with *C. difficile* and plating at 48 h of growth. Data are combined from four independent experiments (*n* = 3–4 biological replicates per condition). Kruskal-Wallis one-way ANOVA with Dunn’s correction vs 0% glucose.

The inhibition of *C. difficile* growth described above could be the result of an inhibitory factor produced by VRE or it could be due to nutrient limitation. To differentiate between these two mechanisms, we first generated VRE conditioned SM in a range of glucose concentrations. Then we created a dilution series of those conditioned media in sterile PBS. VRE conditioned media inhibits *C. difficile* growth with 0.4% and 0.6% total glucose concentration (Fig. 2). When VRE conditioned media containing 0.4% glucose is diluted 1:2 in PBS, it no longer inhibits *C. difficile* growth. These data confirm that VRE inhibits *C. difficile* in a manner consistent with the production of organic acids and not via nutrient limitation.

**Fig 3:**
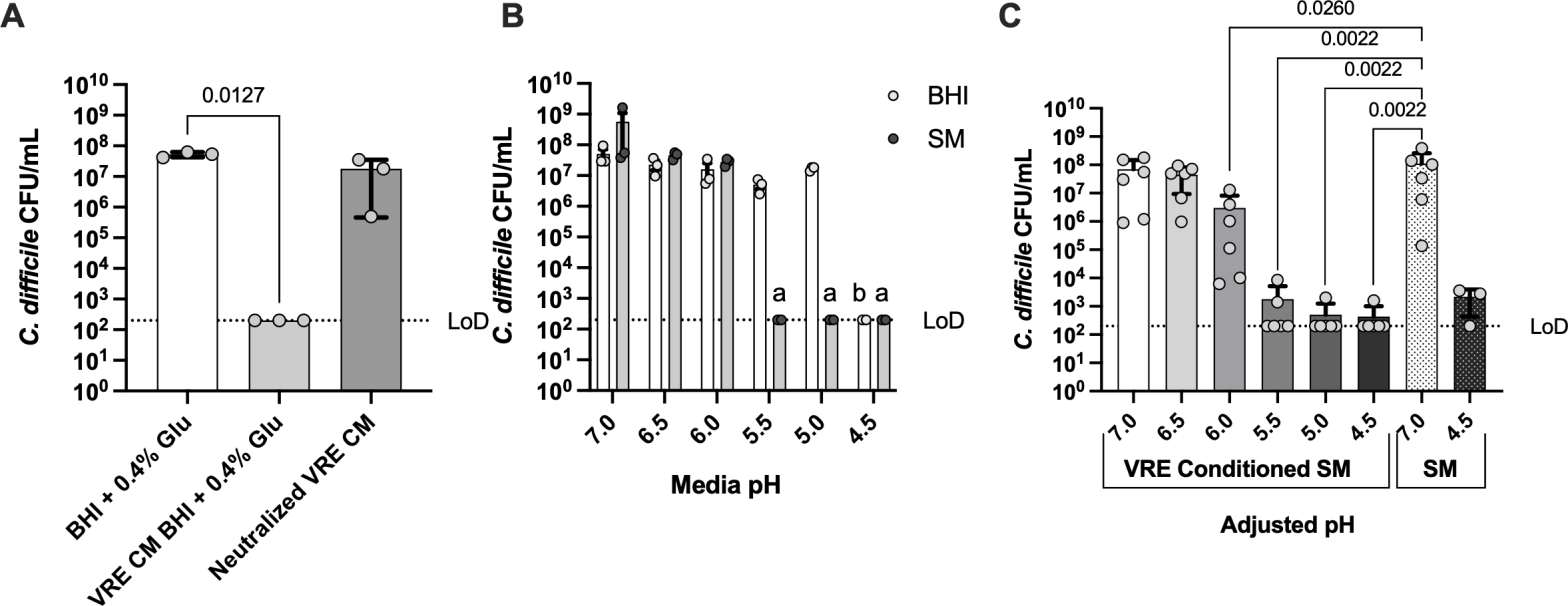
Acidification is necessary and sufficient for inhibition of *C. difficile* (**A**) VRE-conditioned BHI + 0.4% glucose or neutralized with NaOH to a pH of 7 and inoculated with *C. difficile* for 48 h before plating and compared to *C. difficile* growth in naive medium at a pH of 7.0. Data are combined from three independent experiments, *n* = 3 biological replicates, Kruskal-Wallis one-way ANOVA with Dunn’s correction vs. BHI + 0.4% Glu. (**B**) BHI and SM were acidified with reduced HCl in 0.5 unit pH increments before inoculation with *C. difficile* and growth for 48 h. Data are combined from three independent experiments, *n* = 3 biological replicates, a: *P* = 0.0351, b: *P* = 0.0084, Kruskal-Wallis one-way ANOVA with Dunn’s correction versus pH 7.0 CFUs for each medium. (**C**) VRE conditioned SM was filter-sterilized and acidified with reduced HCl before inoculation with *C. difficile* for 48 h. Data are combined from three independent experiments, *n* = 6 biological replicates, Kruskal-Wallis one-way ANOVA with Dunn’s correction versus pH 7.0 SM CFUs for each condition.

To determine if VRE-mediated acidification is necessary for inhibition of *C. difficile,* we neutralized VRE-conditioned BHI + 0.4% glucose (pH 5) with sodium hydroxide to pH 7. In the neutralized conditioned media, *C. difficile* grew to similar levels as in naive BHI + 0.4% glucose (Fig. 3A), suggesting that acidification is necessary for inhibition of *C. difficile* under these conditions. To determine if acidification of media is sufficient to inhibit *C. difficile* growth, we acidified naive BHI and SM (pH 7.0) with hydrochloric acid and found that *C. difficile* growth was inhibited at a pH between 5.0–4.5 in acidified BHI and 6.0–5.5 in acidified SM (Fig. 3B). Finally, to determine if acidification has the potential to synergize with other secreted effectors in VRE conditioned media, we used HCl to acidify VRE-conditioned SM, which had a starting pH of 7.6, to a range of pH from 7.0 to 4.5 Here, we observed an inhibitory pH between 6.0 and 5.5, similar to the threshold observed in HCl acidification of naive SM (Fig. 3C). These data suggest that acidification is the primary inhibitor of *C. difficile* under these *in vitro* conditions.

### VRE can metabolize specific carbon sources into organic acid to inhibit *C. difficile*

**Fig 4:**
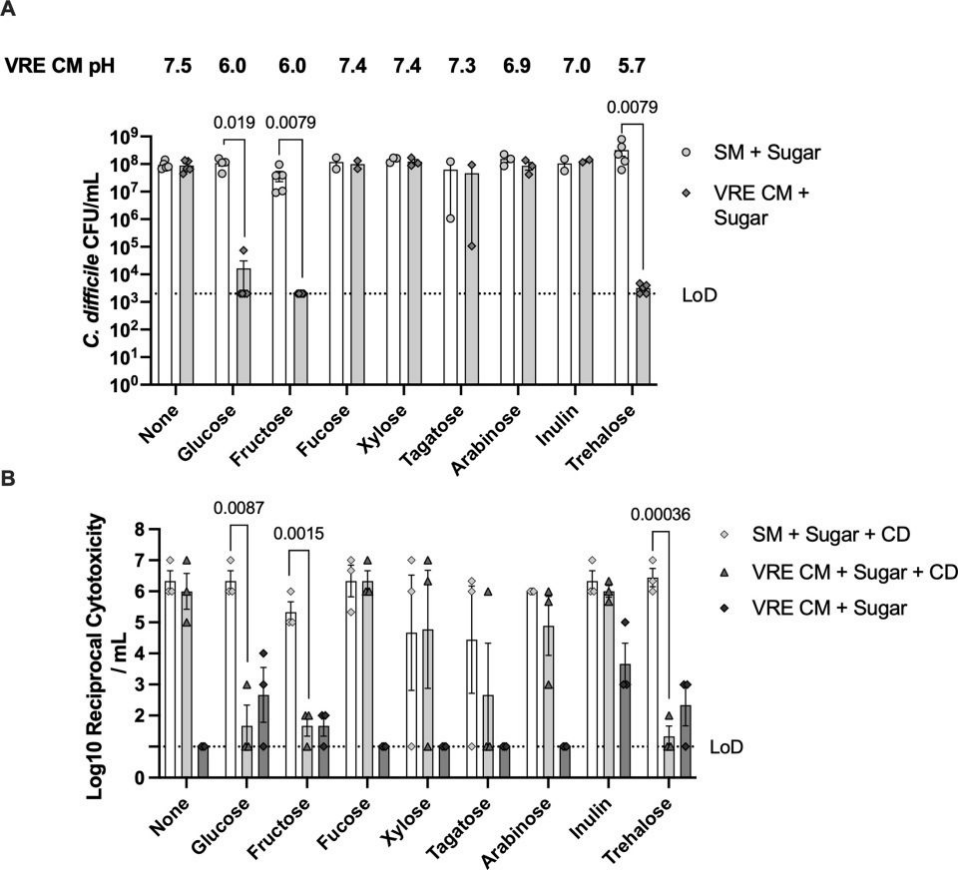
Inhibition of *C. difficile* by VRE is carbon source dependent. (**A**) *C. difficile* was grown in fresh SM + 0.6% of the carbon source (SM + Sugar) indicated on the x-axis or VRE conditioned SM + 0.6% carbon source (VRE CM + Sugar). Starting mean pH of VRE CM + Sugar for each condition is reported above the graph. Data are combined from three to five independent experiments per condition, Mann-Whitney test. (**B**) Cytotoxicity on Vero cells of filtered supernatants from A. Data are representative of two independent assays, Kruskal-Wallis one-way ANOVA with Dunn’s correction versus SM + Sugar + CD for each condition.

Next, we tested the hypothesis that the acidification of any sugar is sufficient to cause VRE to inhibit the growth of *C. difficile* in conditioned media. We used a panel of simple sugars that differed in their reported metabolism by enterococci. Glucose and fructose were reported to be acidified, while fucose and xylose were reported not to be acidified (29). Tagatose and arabinose acidification was reported to differentiate between *E. faecalis* and *E. faecium* (29). We also included the disaccharide trehalose and the polysaccharide inulin, which have been implicated experimentally in mouse models of *C. difficile* infection (30, 31). We generated VRE-conditioned SM with 0.6% of each sugar and grew *C. difficile* for 48 h before plating to simulate biofilm culture conditions. We found that only VRE-conditioned SM containing glucose, fructose, and trehalose inhibited growth of *C. difficile* when compared with growth in the same naive media (Fig. 4A). Glucose, fructose, and trehalose lowered pH of the conditioned media significantly with mean pH of 6.1, 6.0, and 5.7, respectively. These pH levels are consistent with the inhibitory pH levels described above.

We performed cytotoxicity assays on Vero cells using filtered supernatants from the carbon source screen described above. We found significant reductions in cytotoxicity of VRE-conditioned SM containing the acidified carbon sources, glucose, fructose, or trehalose, which reflects the killing of *C. difficile* by VRE (Fig. 4B). It is important to note that although glucose can suppress toxin production via catabolite repression and the transcription factor CcpA during exponential phase (32–34), we did not observe a decrease in cytotoxicity in samples containing *C. difficile* grown in naive SM + 0.6% glucose compared with SM with no carbon source. Our samples were collected at 48 h post-inoculation, well beyond the exponential phase and likely reflecting the total accumulation of toxin over 48 h of growth. Additionally, in supernatants collected from VRE monocultures, we observed an unexpected but statistically significant increase in cytotoxicity from VRE conditioned SM + 0.6% inulin. This cytotoxicity was not neutralized by *C. difficile* anti-toxin.

**Fig 5:**
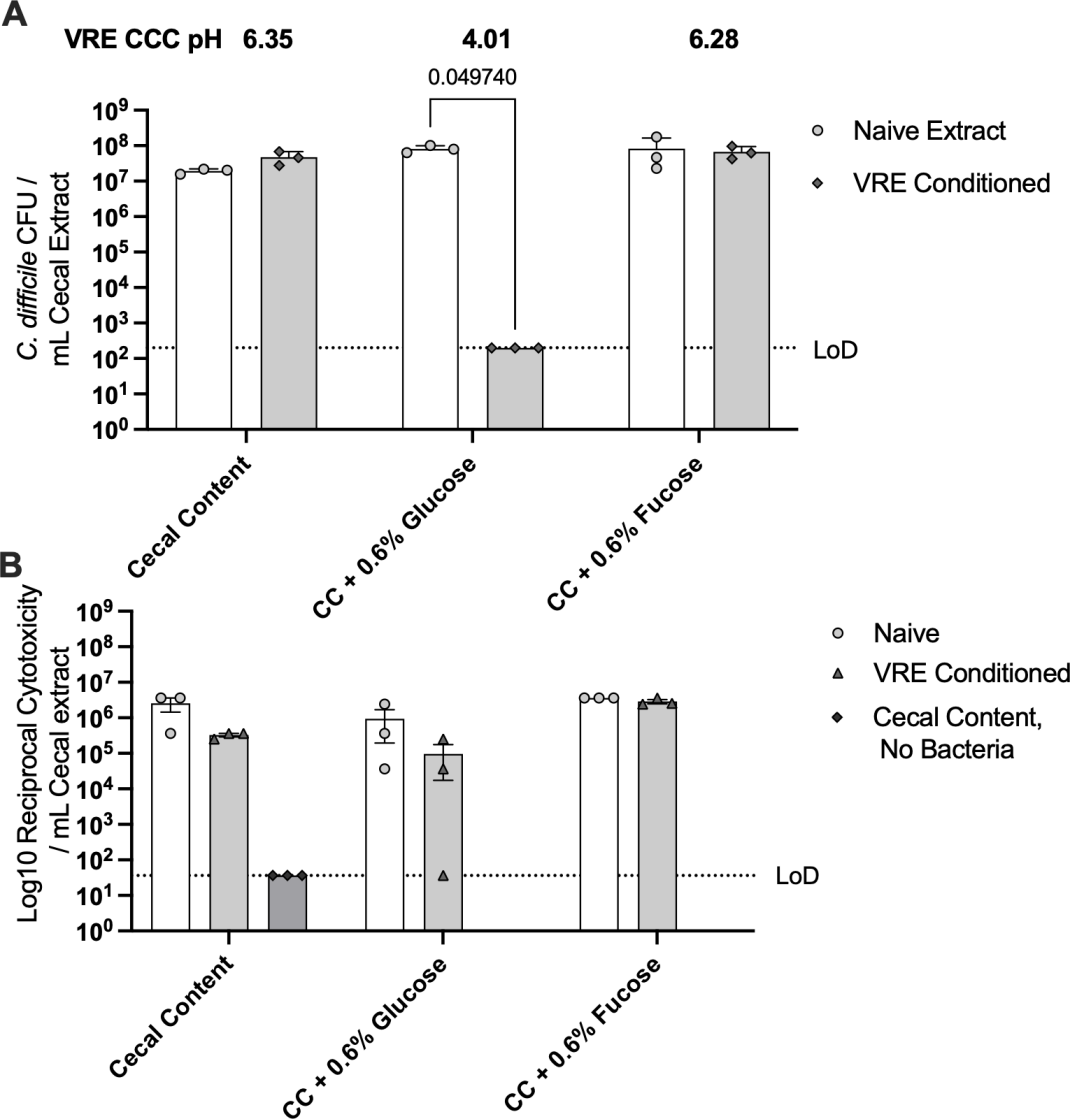
Acidification of glucose inhibits *C. difficile* growth in germ-free cecal content extracts. (**A**) Sterile filtered extracts of cecal content from germ-free mice were prepared with reduced PBS and the indicated added carbon source or equivalent volume PBS and inoculated with VRE and grown for 24 h. Conditioned cecal content (VRE CCC) was sterile filtered then inoculated with *C. difficile* and grown for 48 h followed by plating on selective media. Data are combined from three independent experiments, *n* = 3 biological replicates. Unpaired Welch’s *t*-test. (**B**) Vero cell toxin titer assay of filtered supernatants following *C. difficile* culture. Data are representative of two independent experiments, Unpaired Welch’s *t*-test.

To test the effects of carbon source availability in a more physiologically relevant *in vitro* system, we utilized extracts from cecal content of germ-free mice and compared growth of *C. difficile* in naive and VRE-conditioned filter-sterilized cecal extracts. In cecal content conditioned by VRE, we observed a slight but not statistically significant (*P* = 0.13531) increase in *C. difficile* growth after 48 h (Fig. 5A), which agrees with recent findings that *E. faecalis* can promote *C. difficile* growth *in vivo* (10). However, when we added 0.6% glucose as a carbon source to the cecal extract before VRE conditioning, growth of *C. difficile* was not supported, and pH was reduced to 4.01. Addition of 0.6% fucose, which is not acidified by VRE, as a carbon source did not result in a significant difference in *C. difficile* growth or pH (6.28) between naive and VRE-conditioned cecal extract. These data suggest that the byproducts of carbon source metabolism by VRE can suppress *C. difficile* growth in cecal extracts. In these conditions, we did not observe a significant reduction in cytotoxicity of VRE conditioned cecal content extract containing glucose, despite suppression of *C. difficile* growth below the level of detection (Fig. 5B).

**Fig 6:**
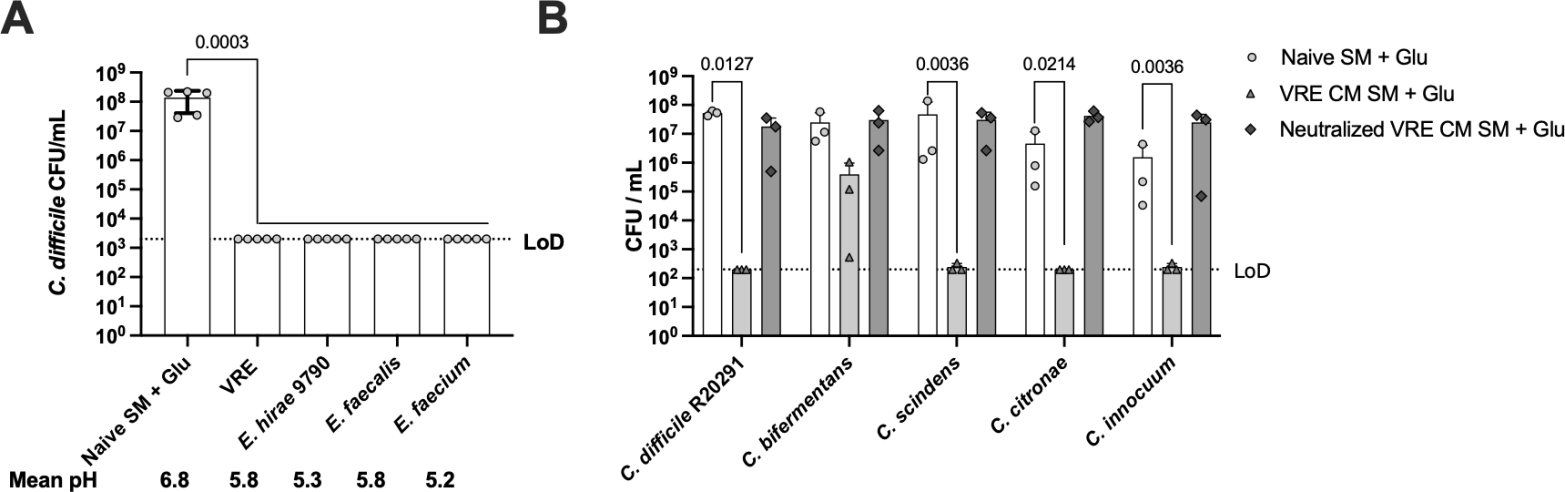
Acidification-mediated inhibition is conserved among enterococci and clostridia. (**A**) SM + 0.6% glucose was conditioned by incubation with the indicated strains on the x-axis followed by inoculation with *C. difficile* and growth for 48 h. Mean pH of the conditioned media is displayed below the x-axis (*n* = 3). CFU data are combined from three independent experiments, *n* = 5 biological replicates, Kruskal-Wallis one-way ANOVA with Dunn’s correction vs Naïve SM + Glu. (**B**) VRE-conditioned SM + 0.6% glucose was created by 48 h of VRE growth followed by filter sterilization or was neutralized with NaOH to pH 7 before filter sterilization and inoculation with *C. difficile* for 48 h. Data are combined from three independent experiments, *n* = 3 biological replicates, Kruskal-Wallis one-way ANOVA with Dunn’s correction vs Naive SM + Glu.

We then tested if acidification-mediated suppression of *C. difficile* is conserved among enterococci. We generated conditioned SM + 0.6% glucose from a panel of enterococci including *E. hirae*, and vancomycin-sensitive isolates of *E. faecium* and *E. faecalis*. All of the tested strains completely suppressed *C. difficile* growth and produced conditioned media with a pH below 6 (Fig. 6A). We then tested a small panel of *Clostridioides* species for growth in VRE conditioned media containing glucose (Fig. 6B). Growth of *C. scindens, C. innocuum, C. citrone, and C. difficile* ribotype 027 strain R20291 was inhibited below the limit of detection, while *C. bifermentans* showed significantly reduced growth. Acidification was necessary for growth inhibition of all strains as growth was restored when VRE conditioned media containing glucose was neutralized with NaOH. These data suggest that acidification is conserved among enterococci and is necessary and sufficient to inhibit growth of diverse *Clostridioides* species under these conditions.

### Fructose supplementation is not sufficient to restore colonization resistance in mice

**Fig 7:**
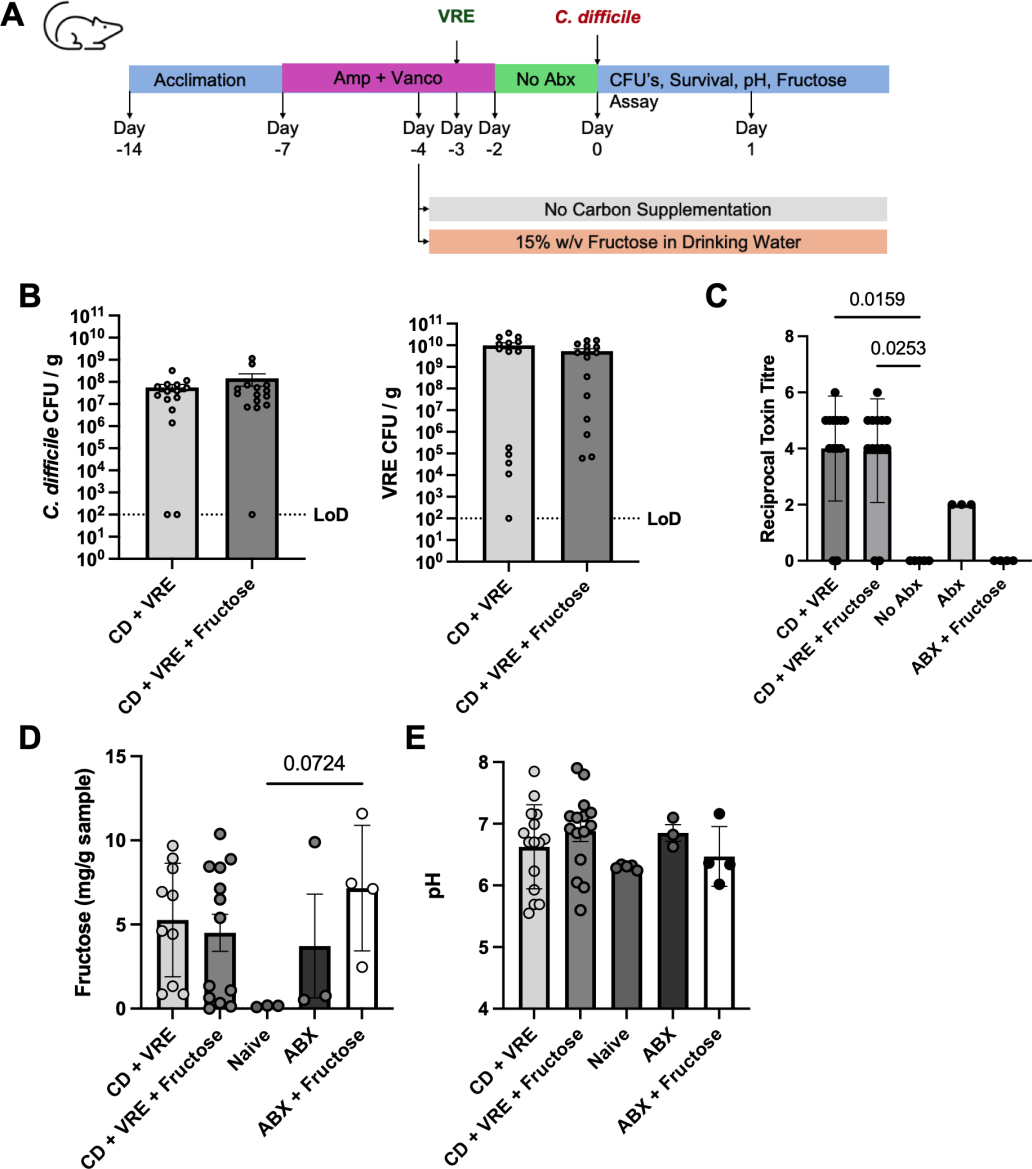
*C. difficile*–VRE dual infection with fructose supplementation. (**A**) Mice were treated with ampicillin and vancomycin in drinking water, and 15% fructose was added to the drinking water of one arm at day −4, followed by VRE colonization on day −3. Antibiotics were withdrawn for 48 h followed by *C. difficile* infection on day 0. Data in B-E are combined from three independent experiments. (**B**) *C. difficile* and VRE CFU levels in cecal content measured by selective plating. (**C**) Vero cell cytotoxicity of filtered cecal content extracts. (**D**) Fructose levels in cecal content measured by an enzymatic assay. (**E**) pH of cecal content. Data were collected with 4–5 mice per condition combined from three independent experiments. Statistics: A and B, *t*-test with Welch’s correction. C–E, one-way ANOVA with Dunn’s correction.

To model the effects of supplementation with a fermentable carbon source, we adapted an existing mouse model by supplementing with 15% w/v fructose in drinking water during *C. difficile* and VRE co-infection (16). Excess dietary fructose accumulates in the colon and alters the metabolism of VRE (35, 36). Mice were sensitized to infection through vancomycin and ampicillin in drinking water, followed by colonization with VRE, which is resistant to both antibiotics (16). Fructose supplementation was added 24 h before the VRE challenge and maintained to the conclusion of the experiment (Fig. 7A). Twenty-four hours after colonization with VRE, antibiotics were withdrawn for 48 h, followed by *C. difficile* infection for 24 h followed by sampling of cecal content. Contrary to our hypothesis, supplementation with fructose in VRE-colonized mice did not affect *C. difficile* colonization or toxin production (Fig. 7B and C). There was a not significant trend toward higher fructose in fructose-supplemented control mice (*P* = 0.07) when compared with naïve mice. However, we did not detect a significant change in fructose levels between CD + VRE mice and CD + VRE + Fructose. We also did not detect a significant change in pH of cecal content between dual-colonized mice and dual-colonized mice supplemented with fructose. We did, however, detect a rise in the mean pH between naïve and antibiotics-treated mice of 6.30 ± 0.02 to 6.85 ± 0.14, as has been reported previously (37). These data suggest that the dietary supplementation attempted here was not sufficient to alter the pH of the cecum.

## DISCUSSION

We initially set out to develop an *in vitro* co-culture biofilm model of VRE and *C. difficile* using excess glucose to promote adherence of the bacteria to plastic plates (38). Growth of both species in excess glucose was not supported in co-culture (Fig. 1). We found that acidification of glucose is necessary, sufficient, and appears to be the primary mechanism by which VRE affects *C. difficile* growth in the presence of excess glucose *in vitro* (Fig. 3). To test if VRE conditioned media inhibits *C. difficile* under all growth conditions, we altered the available carbon source and found that growth inhibition is limited to contexts in which the carbon source is acidified by VRE. We observed a pH-mediated inhibition of growth in the presence of glucose, fructose, and trehalose (Fig. 4A). Added glucose also led to pH-mediated growth inhibition when the growth medium was germ-free mouse cecal content extract (Fig. 5A). These data suggest that control of the available carbon source is critical to developing *in vitro* co-culture assays with lactic acid bacteria. We also measured the toxin titer of *C. difficile* in the carbon source screen and did not find significant changes in toxin production without an underlying inhibition of growth (Fig. 4B). This is likely due to the 48-h time point chosen for analysis, which is beyond the exponential phase in which toxin production is affected by carbon source in single species culture (32). Given the importance of growth phase to toxin production, it is likely that competition for nutrients in dual-species culture may affect the induction of toxin at earlier time points (10).

We also detected what is potentially a novel toxicity of VRE toward cultured Vero cells. This cytotoxic activity was present when conditioned medium was prepared from SM containing 0.6% inulin, a fructan dietary fiber. The samples used in the assay were centrifuged and filtered with a 0.2-μm filter, suggesting that the cytotoxic substance is soluble and filterable. Inulin has generally enhanced protection in human fecal chemostat (39) and mouse models of *C. difficile* infection (30). If inulin induces cytotoxicity by VRE, it is possible that supplementation would result in increased virulence in co-infected mice.

In mouse models of infection, *C. difficile* first begins to accumulate in the cecum 6–12 h post-infection (40). The colon, particularly the cecum, is densely colonized and is a site of high degrees of population diversity and countless metabolic products. A general feature of the mouse (37, 41) and human cecum (42, 43) is an acidic pH of 5.0–6.5 at steady state (44). During *in vitro* culture, *C. difficile* growth and sporulation are limited, following as little as a half-unit shift in pH (45). Growth, sporulation, and germination are almost completely inhibited at a pH of less than 6 (45–47). Treatment with antibiotics in mice raises colon pH from acidic to slightly alkaline conditions, suggesting that the gut microbiota are necessary for maintaining acidic pH (37, 41). These data suggest that the pH of the gut lumen, unlike the pH of blood, is not tightly buffered and may be among the many factors that contribute to *C. difficile* colonization resistance mediated by the gut microbiota. Most bacteria are capable of living in a pH range of 3–4 units, and pH may act as a constraint on growth of commensals and opportunistic pathogens (48). We found that pH-mediated inhibition of *C. difficile* is at least partly bactericidal (Fig. S3). Given that ingested *C. difficile* spores germinate well in the pH neutral small intestine (40), it is possible that transit into the acidic colon (pH ~6) is a natural restraint on growth and contributes to colonization resistance. In humans, a small study found a significant association between an alkaline fecal pH and *C. difficile* infection (49).

The pH of the gut lumen is modulated and constrained by inputs from the host diet, and the metabolism of the gut microbiota and the colonic epithelium. The colonic epithelium produces and secretes bicarbonate, which increases the alkalinity of the gut lumen (50), which could potentially buffer acid production by VRE in our model. However, several studies have noted decreased levels of the bicarbonate transporter DRA (SLC26A3) in the colon of *C. difficile*-infected mice (51–53). In addition to DRA, toxin-dependent damage also reduced the expression of the host glucose transporter SGLT1 in mice and resulted in an increase in the level of glucose in the stool (51). The colonic epithelium also produces lactic acid as a byproduct of glycolysis, which can be converted into short-chain fatty acids by the gut microbiota (54). On the bacteria side of this interaction, in cells of *C. difficile* during active infection, toxin-mediated damage caused the upregulation of carbohydrate PTS importers (55). Carbohydrate metabolism transcripts as a class, including genes involved in fructose metabolism, were increased by both *C. difficile* and *E. faecalis* OG1RF during co-culture *in vitro* (10). Taken together, these data suggest that *C. difficile* and VRE may import and metabolize excess fructose in the colon. Primary metabolism by the gut microbiota produces weak organic acids, such as lactic acid and short-chain fatty acids, when grown in a fermentable carbon source (56). The short-chain fatty acid butyrate is growth suppressive to *C. difficile in vitro* and is inversely correlated with colonization in mice and humans (57, 58). All these factors together have the capacity to influence the standing pH of the large intestine.

In certain cases, such as in critically ill patients treated with antibiotics who become dominated by VRE (4), it may be desirable to enhance the acidity of the gut lumen through administration of a diet or prebiotics that results in acid production and suppression of *C. difficile*. Our data suggest that supplementation with fructose in drinking water alone was not sufficient to affect *C. difficile* colonization in VRE co-infected mice nor was it sufficient to affect the pH of the contents of the cecal lumen (Fig. 7). It is possible that more complex formulations of dietary input (30, 59) may be necessary to restore *C. difficile* colonization resistance in a low-diversity gut microbiota.

Clinical trials have tested the efficacy of probiotic lactic acid bacteria on *C. difficile* infections with mixed results (60, 61). It is clear, however, that strains from genus *Bifidobacterium* and *Lactobacillus* can inhibit *C. difficile in vitro* by decreasing pH (62) and in some cases similar probiotics have reduced *C. difficile* virulence in animal models of infection (63). For example, lactic acid production by *Streptococcus thermophilus* lowered the pH of conditioned media and was inversely correlated with *C. difficile* growth and toxin production *in vitro* and in a mouse model of infection (64). These data suggest that acidification-mediated effects on growth of *C. difficile* are not limited to enterococci and may be a general feature of lactic acid bacteria and any bacteria capable of acidifying a carbon source.

Multiple groups have reported that changes in pH affect growth and survival of core taxa of the gut microbiota. For example, during *in vitro* culture at pH 5.5, multiple human gut isolates from genus *Bacteroides* failed to grow (65). This pH sensitivity of *Bacteroides* has been replicated in several batch culture fermenter studies seeded with donor human feces (66–68). These studies also consistently reported reductions in levels of genus *Clostridioides* (or *Clostridium*) under low pH conditions. In keeping with the batch fermentation studies mentioned above, we also found that commensal *Clostridioides* species are subject to a similar pH-mediated sensitivity to VRE-conditioned media as *C. difficile* (Fig. 6). Therefore, efforts to remediate dysbiosis in recurrent *C. difficile* infection by reintroducing commensal spore-forming anaerobes (69, 70) may benefit from a different strategy. Here, temporarily limiting acid production by enterococci and other lactic acid bacteria through dietary intervention may favor engraftment of the bacteriotherapy. The conundrum here is similar to that faced by chemists since the beginning of antibiotics development: How do you specifically target pathogenic bacteria, while sparing or promoting the closely related commensal strains necessary for steady-state health? A solution will require a more complete knowledge of the metabolism of pathogenic and commensal clostridia.

## MATERIALS AND METHODS

### Strains and growth conditions

Strains are listed in Table S1. All experiments were conducted in an anaerobic chamber (Coy Laboratory Products), with an atmosphere of 90% N_2_, 5% CO_2_ and 5% H_2_. *C. difficile* and other clostridia strains were routinely cultured on BHI plates and liquid media (Brain Heart Infusion + 0.5% yeast extract) supplemented with 0.1% taurocholate and 0.3% L-cysteine. VRE and other enterococci were routinely cultured in BHI liquid medium and Enterococcosel (BD Biosciences) plates. For differential selection during co-culture, VRE and enterococci were plated on Enterococcosel (containing 8 μg/mL vancomycin and 100 μg/mL ampicillin for VRE), while *C. difficile* was plated on BHI plates supplemented with 0.1% taurocholate, 0.3% L-cysteine, 250 μg/mL D-cycloserine, and 16 μg/mL cefoxitin. Commensal *Clostridia* were enumerated by plating on Columbia Blood Agar and counting by distinguishing between colony morphology of VRE.

### Media

BHI was made with 36 g/L Bacto BHI powder with 5 g/L yeast extract, bacterial, autoclaved, then 3-mL filter sterilized 10% L-cysteine was added to the media before aliquoting and reducing in the anaerobic chamber. Sporulation medium was made with 90 g Bacto peptone, 5 g Bacto protease peptone, 1 g ammonium sulfate, and 1.5 g tris base, then 3 mL 10% L-cystine was added after autoclaving. All liquid and plate media were pH adjusted to 7.0 with HCl, unless otherwise specified before autoclaving. Glucose and other sugars used were dissolved into water at a 6% weight per volume ratio before being filter sterilized. Sugars were then added into the media before being placed in the anaerobic chamber or allowed to reduce in the chamber before being added to the media. Inulin was added as described with heating to allow full solubility before being filtered. All plates were poured in 18 mL volumes and kept at 4°C until needed. Then, 8–24 h before using, plates were placed in the anaerobic chamber to reduce fully.

### *C. difficile* growth in VRE and enterococci conditioned media

Mid-log VRE overnight culture was OD-normalized to 0.5 before being inoculated (1:20 dilution) into SM + 0.6% sugar. The culture was grown for at least 24 h anaerobically at 37°C until the OD_600_ was >2 to ensure growth to saturation. Cultures were spun at 1,500 × *g* × 15 min before being filter sterilized with a 0.22-μm filter syringe into 3-mL aliquots into glass tubes. Then, 1.5-mL aliquots were also frozen at −80°C for further use in cytotoxicity assays. *C. difficile* was inoculated 1:60 from mid-log into the VRE-conditioned media and incubated at 37°C for 48 h before being serially diluted and drop plated for CFU/mL enumeration.

### Glucose titration in BHI and SM

BHI and SM were used to create media containing the following glucose concentrations: 0.0%, 0.2%, 0.4% and 0.6%. Media were placed in the anaerobic chamber to reduce before using. VRE was inoculated 1:20 into each glucose concentration of both BHI and SM. Cultures were incubated for 24 h before being filtered sterilized as described above. *C. difficile* was inoculated 1:60 into VRE-conditioned media and fresh BHI and SM conditions and incubated for 24 h before serial dilutions and drop plating for enumeration.

### Dilution of inhibition by VRE-conditioned media

VRE-conditioned media were prepared by inoculating VRE 1:20 into BHI with 0.0%, 0.2%, 0.4%, and 0.6% added glucose. Cultures were grown for 24–36 h to an OD_600_ >2 before being spun down and filter sterilized. A subset of the supernatant was taken out of the chamber for pH recording. The VRE-conditioned media were then diluted 0, 1:1, 1:2, 1:4, and 1:8 with sterile PBS. After dilution, 3-mL aliquots were divided into test tubes per each biological replicate. *C. difficile* mid-log cultures were diluted 1:10 in sporulation media, then added to the aliquots in a 1:60 dilution and grown between 24 h before drop plating for enumeration.

### Acidification and neutralization of SM and BHI

Acidification of SM and BHI was created by titrating HCl into the media to a pH of 7.0, 6.5, 6.0, 5.5, 5.0, and 4.5 before being autoclaved and aliquoted. *C. difficile* was inoculated into the reduced media 1:60 and incubated at 37°C for 48 h before being serially diluted and drop plated for enumeration.

Neutralization was performed by growing VRE in SM + 0.6% glucose or BHI + 0.4% glucose to exhaustion before being spun down and filter sterilized. The filtered media pH was recorded to ensure full acidification at or below 5.5, then neutralized to a pH of 7.0 with NaOH. Neutralized media were filter sterilized again and aliquoted into 3-mL test tubes, then placed in an anaerobic incubator for 24 h to ensure full media reduction. Then, 3-mL aliquots of the acidified media were collected to be used as a negative control. After being reduced, *C. difficile* was inoculated 1:60 into the media conditions. *C. difficile* was also inoculated 1:60 into fresh media at a pH of 7.0 as a positive control. All media conditions were incubated at 37°C for 48 h before drop plating and enumeration.

### Cecal extract *ex vivo* culture

Cecal content from adult germ-free C57BL/6 mice, 12–16 weeks old, was harvested under sterile conditions and frozen at −80C until use. Mice were maintained at an AAALAC-accredited facility under an animal protocol approved by the Institutional Animal Care and Use Committee of Boehringer-Ingelheim Pharmaceuticals Inc. Cecal content extracts were prepared at 0.1 g/mL of wet weight in reduced PBS in an anaerobic chamber. Cecal content extracts were prepared at 0.1 g/mL of wet weight in reduced PBS in an anaerobic chamber. Contents were vortexed and then centrifuged at 1,500 × *g* for 15 min before filter sterilization with a 0.2-μm filter. The extract was split into three conditions with the following supplementation: 0.6% of PBS, glucose, or fucose. VRE was inoculated 1:20 into half of the conditions described, grown for 24 h, spun down, and filter sterilized to create VRE conditioned extract. Mid-log *C. difficile* was inoculated 1:60 from an inoculum into naïve extract or VRE conditioned extract and grown for 24 h at 37°C before serial dilution and drop plating.

### Cytotoxicity assays

Vero cells were grown at 10,000 cells per well in a 96-well plate in Eagle’s minimum essential media (EMEM) + 10% Fetal Bovine Serum and 1× penicillin and streptomycin overnight. Culture supernatants and mice samples were frozen at −80C until assay. All samples were spun down for 15 min at 15,000 × *g* before being filter sterilized into new tubes. Each sample was serially diluted down to 10^−6^ in a fresh 96-well plate with PBS. *C. difficile* purified toxin (TechLab) was diluted into 1 mL of sterile water as a positive toxin control. Then, 96 µL of each sample was placed in the top row of a 96-well plate, and 4 µL of the anti-toxin (TechLab) was added and incubated for at least 20 min to ensure full toxin neutralization from the anti-toxin. Samples with antitoxin were serially diluted in the remaining rows down to 10^−6^ in PBS. Once all toxin, aliquoted samples, and antitoxin samples were completed, 100 µL of each sample and the dilutions was placed onto their respective Vero cell wells. Vero cells were incubated at 37°C with 5.0% CO_2_ overnight. Cytotoxicity was then analyzed with microscopy. Cells that showed rounding were positive for cytotoxicity. To calculate toxin titer, dilutions with less than 80% cell rounding were considered negative, and the previous dilution in the series was considered positive. This dilution number was then used to calculate the Log10 reciprocal toxin titer.

### Conservation of enterococci inhibition of clostridia through acidification

We first grew each *Enterococcus* strain in BHI+0.6% glucose overnight, filter sterilized, and inoculated *C. difficile* to grow for 48 h at 37°C before serial dilution and drop plating on *C. difficile* plates for enumeration. We then used VRE to condition SM + 0.6% glucose, spun down, and filter sterilized as previously described. We then inoculated mid-log cultures of the remaining clostridia panel into the VRE-conditioned media in a 1:60 dilution and grew *C. difficile* for 48 h at 37°C before drop plating. We also performed neutralization and acidification as described above with this clostridia panel.

### Buffering of VRE CM in SM + 0.6% glucose

VRE was grown overnight to an OD of >1.8 in SM or SM + 0.6% glucose with the following buffers; 100 mM PIPES, 100 mM HEPES, 100 mM MOPS. After VRE growth, the samples were plated on enterococcus selective media, the samples were spun down and filter sterilized. An aliquot from each condition was taken to record pH. *C. difficile* was inoculated 1:10 into each VRE-conditioned media and a naive set of conditions. *C. difficile* was grown for 48 h before selectively plating.

### Mouse co-infection

C57BL6 mice, 7 weeks old, were purchased from Jackson Laboratories and were maintained under an approved protocol of the Binghamton University Institutional Animal Care and Use Committee (21-852). Mice were screened for *C. difficile* upon receipt by enrichment culture in CC-BHIS-TA medium (71). Mice were acclimatized for 1 week prior to antibiotics treatment and were maintained in sterile disposable individually ventilated caging with sterile bedding and irradiated food (LabDiet Rodent Diet 20). Mice were treated with 500 mg/L ampicillin + 250 mg/L vancomycin in drinking water for 4 days, switched to water without antibiotics for 48 h followed by infection with 2,500 CFU of *C. difficile* VPI-10463 by oral gavage. Mice receiving VRE were gavaged with VRE after 3 days of antibiotics treatment. For mice receiving dietary supplementation, 15% fructose was added to drinking water after 3 days of antibiotics treatment. Samples were collected and transferred immediately into a pre-reduced anaerobic jar (AnareoPack-Anaero) to minimize oxidative damage to vegetative *C. difficile* during sampling and transport.

### Enumerating bacteria of mouse models

Fecal pellets were collected from mice into sterile, pre-weighed microcentrifuge tubes on the day of acclimation, the day of VRE challenge, the day of *C. difficile* challenge, and 24 h after. Commensal enterococci were plated on non-selective enterococci plates and were maintained through the duration of the experiments. Twenty-four hours after *C. difficile* challenge, mice were euthanized using an Euthanix lid for 10 min before cervical dislocation. Cecal content was collected into two tubes, one for CFU plating and one for pH measurements. To calculate CFU’s per 1 g sample, tubes were weighed before and after sample collections, and the raw CFUs were divided by the sample weight. During *C. difficile* infection, tubes were placed in an anaerobic box before collecting. Samples were quickly collected from each mouse and put back into the anaerobic box before processing. All samples were resuspended in 1.0 mL pre-reduced PBS before plating. Samples were frozen at −80°C until further use after initial CFU plating. Cecal pH was collected by dissolving cecal contents in 200 µL nanopure water before pH measurement using an Accumet micro-electrode (Fisher).

### Fructose assay

Cecal contents used for CFU plating and cytotoxicity assays were used for fructose concentration analysis by a colorimetric assay kit (NovusBio). The protocol was followed with the following adaptations: a standard curve was created using 0, 500, 800, 1,000, 1,600, 1,800, and 2,000 µg/mL fructose, and the linear equation was calculated as y = 0.0002x + 0.0831. Then, 25 µL of sample was combined with 1.5 mL assay kit solution and boiled for 8 min at 100°C. A Nanodrop (Thermo-Fisher) was blanked with nanopure water and measured at 285 nm to analyze standard values and all sample values.

## SUPPLEMENTAL MATERIAL

Supplemental material iai.00159-26-s0001.docx Figures S1–S4; Table S1.

ASM does not own the copyrights to Supplemental Material that may be linked to, or accessed through, an article. The authors have granted ASM a non-exclusive, world-wide license to publish the Supplemental Material files. Please contact the corresponding author directly for reuse.
